# The Effectiveness of Cefazolin Prophylaxis on Infection after Transureteral Lithotripsy: A Randomized Clinical Trial

**DOI:** 10.4314/ejhs.v33i6.16

**Published:** 2023-11

**Authors:** Mohammad Taheri, Ahmad Kameli, Ramin Haghighi

**Affiliations:** 1 Department of Internal Medicine, Faculty of Medicine, Mashahd University of Medical Sciences, Mashahd, Iran; 2 Department of Nursing, North Khorasan University of Medical Science, Bojnurd, Iran; 3 Department of Urology, Faculty of Medicine, North Khorasan University of Medical Sciences, Bojnurd, Iran

**Keywords:** Transureteral lithotripsy, Prophylaxis, Cefazolin, Ureteral stone

## Abstract

**Background:**

Transureteral lithotripsy (TUL) is one of the most common surgeries in urology, and many TUL procedures have been performed with antibiotics prophylaxis. The present study investigates the effect of antibiotic prophylaxis on the rate of urinary infection after TUL.

**Methods:**

This double-blind, randomized clinical trial was conducted on 158 patients with ureteral stones, with 79 in each group: the prophylaxis cefazolin group (Group A) and the placebo group (Group B). The patients were referred to Imam Hassan Hospital in Bojnurd, Iran. The standard technique of TUL operation was performed using a pneumatic lithoclast and a semirigid 9/8/Fr ureteroscope. The bacterial isolates were identified through growth on EMB agar and blood agar. Antimicrobial sensitivity testing (AST) was carried out by disc diffusion technique.

**Results:**

According to our results, 157 patients were eligible for analysis; 79 patients in Group A and 78 patients in Group B. Flank pain and urinary complaints were the most common symptoms. Our findings indicate that cefazolin prophylaxis did not show any significant differences in preventing postoperative infection between the two groups. E. coli accounted for eight 10.1% (8/79) Group A and 9% (7/78) in Group B, respectively. The results of AST for the 15 E. coli strains revealed a high rate of antibiotic resistance against ampicillin (73.3%).

**Conclusion:**

Our findings indicate that prophylactic antibiotic administration does not demonstrate effectiveness in reducing the infection rate following TUL surgery. Antibiotic prophylaxis is not recommended considering the potential adverse effects, cost implications, risk of antibiotic resistance, and lack of efficacy.

## Introduction

Ureteral stones represent a common urological condition that results in an increasing number of patients being referred to hospital emergency units. The prevalence of ureteral stones has steadily risen over the past few decades, resulting in significant morbidity and healthcare costs (1-3). Timely and effective management of ureterolithiasis is crucial for alleviating symptoms, preventing complications, and improving patient outcomes. One crucial aspect of ureterolithiasis treatment involves the prevention of postoperative infections, which can adversely impact patient recovery and overall prognosis (4). Strategies such as antibiotic prophylaxis, preoperative urinary tract infection (UTI) treatment, and minimizing procedural time have the potential to reduce the risk of infection (5,6).

Among different therapeutic methods, Transureteral lithotripsy (TUL) is the most prevalent surgical modality for ureteral stones (7,8). TUL offers several advantages, including its minimally invasive nature and high success rates in stone fragmentation (9). However, TUL carries the risk of postoperative infections, ranging from localized UTIs to more severe systemic infections, such as sepsis (10).

The etiology of post-TUL infections is multifactorial, including immunocompromised status, comorbidities (e.g., diabetes mellitus), and urinary tract abnormalities that increase susceptibility to infections (11). Procedural factors, including surgery duration, indwelling ureteral stents, infectious stone, and residual stone fragments, can also contribute to development of postoperative infections. Identifying effective strategies to reduce the incidence of infections after TUL is crucial for optimizing patient outcomes and improving the quality of care (7,12,13).

Antibiotic prophylaxis is a widely accepted approach to prevent postoperative infections in various surgical procedures. Prophylactic antibiotic administration aims to reduce the microbial load, minimize the risk of bacterial colonization, and prevent the progression to clinical infection (7) (14). In the context of ureteral stone removal surgery, selecting an appropriate prophylactic antibiotic regimen holds significant clinical importance. Cefazolin, a first-generation cephalosporin, is commonly employed as a prophylactic antibiotic in many surgeries due to its favorable pharmacokinetic properties in preventing surgical site infections (15,16).

Some studies did not recommend prophylaxis for ureteroscopy and reported that antibiotic prophylaxis had no effect on fever and infection after ureteroscopy (16,17). However, some others reported different prophylaxis results in reducing the incidence of UTI in healthy people with urolithiasis (15,18). Despite the widespread use of cefazolin prophylaxis in urological procedures, there remains a lack of consensus regarding its optimal dosage, duration, and effectiveness in reducing postoperative infections, specifically in the context of ureteral stone surgery. Therefore, this study investigates the possible effects of cefazolin prophylaxis on infection rate after TUL in patients receiving cefazolin prophylaxis compared to those receiving placebo.

## Methods and Patients

**Study design and sample size**: This study was a controlled randomized clinical trial with a double-blind design involving 158 patients with ureteral stones referred to Imam Hassan Hospital in Bojnurd, Iran. The patients were divided into two groups: Group A received cefazolin prophylaxis, and Group B received placebo.

All patients provided their consent to participate in the study. All methods were performed following the Declaration of Helsinki, and the study received approval from the Ethical Code Committee (IR.NKUMS.REC.1397,016) and was registered under the IRCT code (IRCT20160514027893N2). Demographical data and clinical characteristics of all the patients were recorded. The sample size calculation was based on the ratio of post-operation bacteriuria between the two groups, with a ratio of 3.5% for patients receiving prophylaxis and 35% for patients not receiving cefazolin prophylaxis. Additionally, pyuria was reported at 49.1% for the prophylaxis group and 22.8% for patients without prophylaxis. The minimum sample size for Cronbach's alpha analysis was evaluated at 75 participants for each group. Finally, according to the type of study and considering the possibility of patient dropout, 79 patients were selected for each group.

**Patients and procedure**: The study included patients with ureteral stones, had a negative result on urine culture (UC) and no fever before the surgery. The exclusion criteria comprised previous history of malignancies, diabetes, immunodeficiency, chemotherapy, infectious diseases, and allergies to cefazolin. Also, patients who recently received prophylaxis endocarditis treatment and antibiotics or required simultaneous surgeries were excluded from the study. Group A received cefazolin prophylaxis at a dose of 1 gram, while, Group B received a normal saline solution as a placebo. The injection of both the placebo and cefazolin occurred less than 2 hours before the operation.

An experienced urologist performed the surgery with the patient in the lithotomy position, following appropriate prep and draping, under either spinal or general anesthesia. Two types of ureteroscopy (URS) were applied: Fr8/9 and Fr6. In cases where children could not accommodate the Fr8/9 ureteroscope or when the Fr8/9 scope could not access the ureter, the Fr6 ureteroscope was utilized. The applied mechanism of breaking the stone was pneumatic. Approximately 48 hours post-TUL, body temperature, UC, and UA were assessed for all patients.

**Variables**: Data on age, gender, weight, duration of the disease, various complaints such as flank pain, urinary symptoms (dysuria, frequency, and urgency), nausea, vomiting, and negative results of UC before surgery were recorded for all patients. Additionally, information on stone types, stone location, diagnostic methods (CT-scan or ultrasonography), prior treatments for stone diseases such as Extracorporeal Shock Wave Lithotripsy (ESWL), Transureteral Lithotripsy (TUL), and Percutaneous Nephrolithotomy (PCNL), types of ureteroscope, duration between symptom onset and surgery, surgery duration, types of anesthesia (regional or general), procedure difficulty level (easy, moderate, or difficult), and need for a double J (DJ) stent were documented. Patients with incomplete data or those who experienced complications during the surgery were excluded from the study

**Bacterial isolates**: The bacterial isolates were identified by growth on Eosin Methylene Blue (EMB) agar and blood agar, Gram-staining reaction, and performing biochemical tests, depending on whether the isolate was Gram-positive or Gram-negative (19). Antimicrobial sensitivity testing (AST) was carried out by modified Kirby Bauer disc diffusion technique on Muller Hinton agar (Oxoid Ltd), and results were interpreted in accordance with Clinical Laboratory Standards Institute guidelines (CLSI). *Escherichia coli* ATCC 25922 was used as control strains for AST (20). For the AST, different antibiotic disk were used as follows: ciprofloxacin (5 µg), gentamycin (10 µg), trimethoprim-sulfamethoxazole (1.25/23.75 µg), ceftriaxone (30 µg), ampicillin (10 µg), meropenem (10 µg), ceftazidime (30 µg), cefepime(30 µg), cefuroxime (30 µg), amikacin (30 µg), and piperacillin-tazobactam (30 µg) (PadTan, Iran).

**Statistical analysis**: The data were reported as mean ± standard deviation, number, and percentage. The Spearman and Pearson correlation coefficients were used to check the relationship between quantitative and qualitative variables. Also, the Independent T-test was used for quantitative variables, and the Chi-square test was used for qualitative variables. All data were analyzed using SPSS version.20, and a significance level of 0.05 was considered in this study.

## Results

A total of 158 patients were enrolled and randomly assigned. One patient was excluded due to pyuria found before surgery. According to our results, 157 were eligible for analysis; 79 patients in group A and 78 patients in group B. Based on baseline characteristics of the patients, 61.1% (96/157) were males, and 32.5 (51/157) had a family history of urothelial stones. The mean age of the patients in group A was 38.03±15.30 years, and in group B was 39.2±17.15 (2 to 81 years), and the mean duration of the disease was 6.03±5.80 years among patients.

The mean weight of the patients was 80.18±18.5 kg/cm2. Among various complications, flunk pain and urinary complaints were the most common complaints among patients before and after surgery, respectively; none presented with a fever. The mean duration from the onset of the patient's symptoms to the TUL procedure was 5.4 days (12 hours to 24 days), and the duration of surgery was 14.62±2.79 minutes (10-20 minutes). Approximately 85% of the patients underwent general anesthesia, ultrasonography being the most frequently used diagnostic method (n=84). Calcium oxalate was the most commonly detected stone type (9.4%).

Most patients had no history of previous surgical treatment for the stones (66.9%, 105/157). The majority of stones were located in the lower ureter, and Fr9/8 was applied in 80.2% (126/157) of the surgeries, with 34 patients requiring a DJ stent. Microscopic hematuria was reported in 87.2% (137/157) patients as a common urinary complication after surgery; ten patients experienced gross hematuria; and three patients had no hematuria. The mean pain score, as reported in the questionnaire, was (4.43±1.57) among adults (Table 2). All patients were discharged from the hospital within 6±0.88 hours. Furthermore, detailed demographic data and clinical characteristics of the patients in both groups are presented in Table 2.

**Table 2:** Comparison of the mean of WBC count and time of discharge among patients

Variables	Groups	Mean±SD		*P value*
WBC count in urine before the surgery	Prophylaxis cefazolin	10.03±2.71		0.765
	Placebo	10.18±2.46		
WBC count in urine after the surgery	Prophylaxis cefazolin	14.45±2.30		0.306
	Placebo	14.03±2.76		
Discharge after surgery	Prophylaxis cefazolin	4.66±0.93		0.062
(Hour)	Placebo	4.33±0.80		

WBC (white blood cell)

**Identified bacterial isolates**: The results of UC demonstrated no statistically significant differences between the two studied groups, with eight isolates in group A and seven isolates in group B testing positive for Gram-negative bacteria (P=0.558). Among the identified bacterial types, *E. coli* accounted for 10.1% (8/79) in group A and 9% (7/78) in group B, respectively. The results of antibiotic susceptibility testing for the 15 *E. coli* strains revealed a high rate of antibiotic resistance, particularly against ampicillin (73.3%), followed by nalidixic acid (60%), ceftriaxone (40%), ceftazidime (40%), cefepime (40%), cefuroxime (40%), gentamycin (33.3%), trimethoprimsulfamethoxazole (26.7%), and ciprofloxacin (26.7%). All isolates were susceptible to meropenem, amikacin, and piperacillintazobactam.

## Discussion

While TUL is considered a clean surgery and is a clean surgical procedure and a common approach for managing ureteral stones, it is not entirely immune to infections. In addition, postoperative infections, particularly fever, and sepsis, have been documented in patients who underwent TUL. Understanding the occurrence and prevalence of these complications is essential for evaluating the safety and efficacy of TUL procedures (21,22). In this study, we aimed to investigate the incidence of complications and the presence of fever among patients undergoing TUL, shedding light on their potential impact on patient outcomes

Fever was used as an indicator of infection during the 24-hour follow-up after surgery. None of the patients in our study met the criteria for fever. Most of our patients had a long history of dealing with ureteral stones, with calcium oxalate being the most commonly detected stone type. The lower ureter was the predominant site of stone localization.

Ye et al. also reported that calcium oxalate was the most frequently detected stone among patients (23). In the current study, flank pain was the most common complaint experienced by patients before surgery, while urgency was the most common complaint after surgery. As previously reported, following urinary tract interventional therapy, many patients represented symptoms such as hematuria, pain or discomfort, dysuria, frequency, urgency, and UTI (24).

Urinary sepsis is a post-ureterorenoscopy complication, particularly in patients with a history of urinary sepsis, previous positive UC, antibiotic therapy, DJ stent, or residual lithiasis (25). Various prophylaxis regimens have been proposed to prevent post-surgical infections, including fluoroquinolones, aminoglycosides, penicillins with beta-lactam inhibitors, and first and second-generation cephalosporins (26). Cefazolin prophylaxis, a first-generation antibiotic, is commonly employed to prevent postoperative infections in patients (27-29). Hsieh et al. reported the effectiveness of cefazolin against both gram-positive and gram-negative microorganisms, including *E. coli* (30). Furthermore, Peng et al. illustrated a high incidence of UTI in patients undergoing retrograde upper urinary lithotripsy (31).

There is no definitive evidence regarding the duration of antibiotic prophylaxis. The guidelines of the EAU, the American Urological Association (AUA), and the Japanese Urological Association (JUA) suggest single-dose prophylaxis. Besides, definitive regimen has been established (32-34).

Our results show that cefazolin prophylaxis did not demonstrate significant differences in preventing postoperative infection between the two groups. It should be considered that the small sample size made limited our ability to identify significant differences. The advantage of our study lies in its real-world practice-based design. In line with our findings, a randomized clinical trial conducted in Iran by Aghamir et al. showed no significant differences between the two groups in terms of operation time, length of hospital stay, postoperative bacteriuria, positive urine culture, postoperative fever, and the overall success rate of TUL. The study indicates that patients undergoing TUL without antibiotic prophylaxis do not experience an increased incidence of infectious complications (35).

Many urologists perform TUL without antibiotic prophylaxis; however, the use of chemoprophylaxis before TUL remains a subject of controversy. Takahashi et al. reported that a single dose of antimicrobial prophylaxis could be effective for patients undergoing TUL(36). Additionally, Knopf et al. demonstrated the potential effectiveness of perioperative prophylaxis in the case of an unexpected intraoperative complication during ureteroscopic stone removal (37). Similar with our findings, some studies reported that antibiotic prophylaxis did not reduce the risk of infectious complications in the intervention group compared to the placebo group (30, 38-40).

In summary, our findings suggest that prophylactic administration of cefazolin does not demonstrate effectiveness in reducing the incidence of postoperative complications (such as fever, UTI, etc.) following TUL. Additionally, it does not increase the incidence of complications in patients undergoing the TUL procedure without prophylaxis. Therefore, we do not recommend antibiotic prophylaxis, considering the potential adverse effects, cost implications, risk of antibiotic resistance, and lack of efficacy. Moreover, *E. coli* isolates were the most prevalent isolates, partially resistant to the tested antibiotics.

## Figures and Tables

**Table 1:** The frequency of demographical data and clinical characteristics of the patients with urolitiasis underwent TUL

Variable		Frequency n (%)		
		Total (n=157)	IY cefazolin (n=79)	no-prophylaxis (n=78)
**Gender**	Male	96 (61.1)	47 (59.5)	49 (62.8)
	Female	61 (38.8)	32 (40.5)	29 (37.2)
**Family history of urothelial**	Yes	51 (32.5)	28 (35.4)	23 (29.5)
	No	106 (67.5)	51 (64.6)	55 (70.5)
**History of previous intervention**	TUL	36 (22.9)	20 (25.3)	16 (20.5)
	OWL	10 (6.4)	5 (6.3)	5 (6.4)
	PCNL	6 (3.8)	2 (2.5)	4 (5.1)
	None	105 (66.9)	52 (65.8)	53 (67.9)
	CT-scan	56 (35.7)	30 (38)	26 (33.3)
**Diagnosis methods**	Ultrasonography	84 (53.5)	40 (50.6)	44 (56.4)
	Both	17 (10.8)	10 (12.7)	7 (90
**Difficulty degree of the TUL**	Easy	139 (88.5)	75 (95)	64 (82)
	Moderate	13 (8.2)	6 (7.6)	7 (9)
	Difficult	5 (3.2)	3 (3.8)	2 (2.6)
	Upper ureter	43 (27.4)	24 (30.4)	19 (24.3)
**Location of the stone**	Middle ureter	32 (20.4)	20 (25.3)	12 (15.4)
	Lower ureter	82 (52.2)	50 (63.2)	32 (41)
**Types of URS**	Fr8/9	126 (80.2)	65 (82.3)	61 (76.2)
	Fr6	21 (19.8)	11 (13.9)	10 (12.8)
**Need for double J stent**	Yes	34 (21.7)	22 (27.8)	12 (15.4)
	No	123 (78.3)	57 (72.2)	66 (84.6)
**Urinary complaints After surgery**	Urinary complaint	87 (55.4)	40 (50.6)	47 (60.3)
	Flunk pain	38 (24.2)	25 (31.6)	13 (16.7)
	Nausea and vomiting	32 (23.4)	20 (25.3)	12 (15.4)
	Microscopic	137 (87.2)	65 (82.3)	72 (92.3)
**Hematuria**	macroscopic	16 (10.2)	8 (10.1)	8 (10.3)
	None	4 (2.6)	2 (2.5)	2 (2.6)

Transureteral lithotripsy (TUL); Extracorporeal shock wave lithotripsy (ESWL); Percutaneous nephrolithotomy (PCNL); * Urinary complaint include frequency, urgency, dysuria

**Table 3:** Antibiotic resistant rate of *E. coli* isolated from urine in patients after performing TUL

Antibiotics	Group A (%)	Group B (%)
Ampicillin	75	71.4
Nalidixic acid	62.5	57.1
Gentamycin	37.5	28.6
Ceftazidime	37.5	42.9
Cefepime	37.5	42.9
Cefuroxime	37.5	42.9
Ceftriaxone	37.5	42.9
Trimethoprim-Sulfamethoxazole	25	28.6
Ciprofloxacin	25	28.6
Amikacin	0	0
Piperacillin-Tazobactam	0	0
Meropenem	0	0
